# Confronting two-pair primer design for enzyme-free SNP genotyping based on a genetic algorithm

**DOI:** 10.1186/1471-2105-11-509

**Published:** 2010-10-13

**Authors:** Cheng-Hong Yang, Yu-Huei Cheng, Li-Yeh Chuang, Hsueh-Wei Chang

**Affiliations:** 1 Department of Electronic Engineering, National Kaohsiung University of Applied Sciences, Kaohsiung, Taiwan; 2 Department of Network Systems, Toko University, Chiayi, Taiwan; 3 Department of Chemical Engineering & Institute of Biotechnology and Chemical Engineering, I-Shou University, Kaohsiung, Taiwan; 4 Department of Biomedical Science and Environmental Biology, Kaohsiung Medical University, Kaohsiung, Taiwan; 5 Graduate Institute of Natural Products, College of Pharmacy, Kaohsiung Medical University, Kaohsiung, Taiwan; 6 Center of Excellence for Environmental Medicine, Kaohsiung Medical University, Kaohsiung, Taiwan; 7 Cancer Center, Kaohsiung Medical University Hospital, Kaohsiung Medical University, Kaohsiung, Taiwan

## Abstract

**Background:**

Polymerase chain reaction with confronting two-pair primers (PCR-CTPP) method produces allele-specific DNA bands of different lengths by adding four designed primers and it achieves the single nucleotide polymorphism (SNP) genotyping by electrophoresis without further steps. It is a time- and cost-effective SNP genotyping method that has the advantage of simplicity. However, computation of feasible CTPP primers is still challenging.

**Results:**

In this study, we propose a GA (genetic algorithm)-based method to design a feasible CTPP primer set to perform a reliable PCR experiment. The SLC6A4 gene was tested with 288 SNPs for dry dock experiments which indicated that the proposed algorithm provides CTPP primers satisfied most primer constraints. One SNP rs12449783 in the SLC6A4 gene was taken as an example for the genotyping experiments using electrophoresis which validated the GA-based design method as providing reliable CTPP primer sets for SNP genotyping.

**Conclusions:**

The GA-based CTPP primer design method provides all forms of estimation for the common primer constraints of PCR-CTPP. The GA-CTPP program is implemented in JAVA and a user-friendly input interface is freely available at http://bio.kuas.edu.tw/ga-ctpp/.

## Background

Genotyping is a common technique used in association studies of diseases and cancers. Although many high-throughput platforms of single nucleotide polymorphism (SNP) genotyping, such as SNP array [1] and real-time PCR using TaqMan probes [2], have been introduced, most laboratories still validate SNP or novel mutation by PCR-restriction fragment length polymorphism (RFLP) genotyping [3-6] because this method is inexpensive for small-scale genotyping. One shortcoming of PCR-RFLP is its long digestion time (usually in 2-3 hours) for restriction enzymes [7,8].

Recently, a restriction enzyme-free SNP genotyping technique called "PCR with confronting two-pair primers (PCR-CTPP)" was developed [9-12]. It has been applied successfully to at least 30 different SNP genotypings. For example, interleukin-1B (IL-1B) C-31T, interleukin-2 (IL-2) -330G, beta2-adrenergic receptor (beta2-AR) Gln27Glu, and aldehyde dehydrogenase 2 (ALDH2) were genotyped by PCR-CTPP for association studies with smoking behavior [13], pylori-induced gastric atrophy [14], severe coronary artery disease [15], and esophageal cancer risk [16], respectively. There is no doubt that the PCR-CTPP method is suitable and reliable for most cases of SNPs. This method considerably lowers the need to consume restriction enzymes. However, the criteria for the PCR-CTPP primers are only tolerant of a small difference in melting temperature (*T*_m-diff_) between the four primers in the PCR-CTPP method [10]. Moreover, typical primer design constraints also need to be considered, such as primer length, primer length difference, PCR product length, GC proportion, melting temperature (*T*_m_), GC clamp, dimer (including cross-dimers and self-dimers), hairpin structure, and specificity. Hence, the computational requirements needed to improve the primer design with PCR-CTTP are rather high.

To design CTPP primers with many corresponding constraints, we introduce a genetic algorithm (GA) [17,18] to improve the design of CTPP primer sets. GA is a stochastic search algorithm modeled on the process of natural selection underlying biological evolution [19]. It constitutes a randomized search and an optimization technique that derives its working principle from natural genetics. Since GA computation is similar in nature to the evolution of the species, it best fits DNA behavior associated with SNP interaction [20] and general primer design [21]. The evolutionary computations involved, such as selection, crossover and mutation, are effective in achieving optimal solutions for many CTPP primer sets. After each run, chromosomes in a GA share information with each other and the superior solutions based on a fitness rule are refined from generation to generation. Therefore, CTPP primers obeying the typical primer design constraints described above can be mined.

## Methods

### Problem formulation

The CTPP primer design problem can be described as follows. Let *T_D _*be a template DNA sequence, which is composed of nucleotide codes with an identified SNP. *T_D _*is defined by:

(1) $$\begin{array}{l} TD=\{Bi|\;i\text{ is the index of DNA sequence,}\\ \text{1}\leq \;i\;\leq \iota ,\;\exists \;!Bi\in \text{IUPAC code of SNP\}} \end{array}$$

where *B_i _*is the regular nucleotide code (A, T, C, or G) mixed with a single IUPAC code of SNP (M, R, W, S, Y, K, V, H, D, B or N) (is the existence and uniqueness). For the target SNP, we focused only on true SNPs described in dbSNP [22] of NCBI, i.e., deletion/insertion polymorphisms (DIPs) and multi-nucleotide polymorphisms (MNPs) are not included.

**Figure 1:**
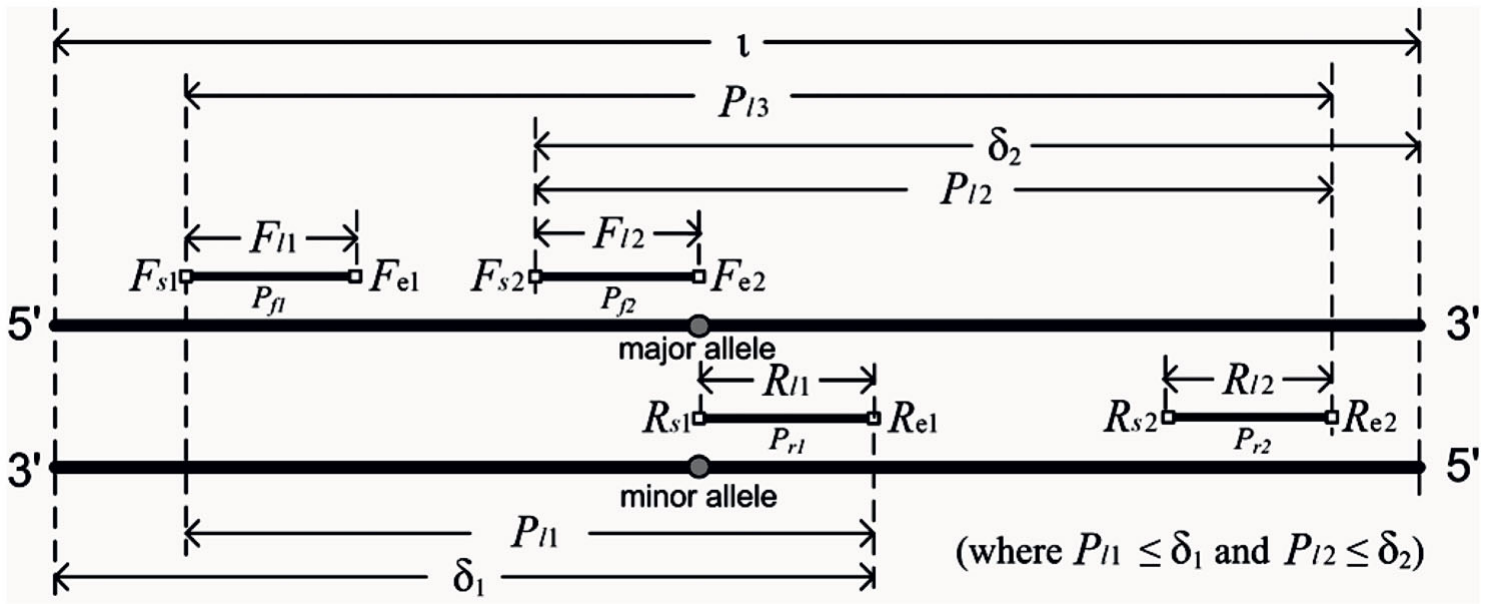
**Parameters of the template DNA and the CTPP primer set**. Symbols indicate: *F*: Forward primer; *R*: Reverse primer; *s*: Start nucleotide position; *e*: End nucleotide position; *P*: Length of PCR product using a primer set (*F*/*R*); *l*: Length of primer or product;ι: Length of DNA template; δ_1_: Length from the *R*_*s*1 _end to downstream of template DNA; δ_2_: Length from *F*_*s*2 _to the downstream end of template DNA.

The CTPP primer design requires two pairs of short sequences which are constraining in *T_D _*based on a defined SNP site as illustrated (Figure 1). The forward primer 1 (*P*_*f*1_) is a short sense sequence in the upstream (5' end) of a defined SNP site for some distances; the reverse primer 1 (*P*_*r*1_) is a short antisense sequence which contains a nucleotide (the minor allele of the defined SNP site) located at its 3' end; the forward primer 2 (*P*_*f*2_) is a short sense sequence which contains a nucleotide (the major allele of the defined SNP site) located at its 3' end; and the reverse primer 2 (*P*_*r*2_) is the antisense sequence in the upstream of a defined SNP site for some distances. These four primers are defined as follows:

(2) $$Pr\text{1}=\{\bar{B_{i}}|i\text{ is the index of }TD\text{, }Rs1\;\leq \;i\;\leq R\text{e1}\}$$

(3) $$Pr\text{1}=\{\bar{B_{i}}|i\text{ is the index of }TD\text{, }Rs1\;\leq \;i\;\leq R\text{e1}\}$$

(4) Pf2={Bi|i is the index of TD, Fs2 ≤ i ≤Fe2}

(5) $$Pr\text{2}=\{\bar{B_{i}}|i\text{ is the index of }TD\text{, }Rs2\;\leq \;i\;\leq R\text{e2}\}$$

where both *P*_*f*1_/*P*_*r*1 _and *P*_*f*2_/*P*_*r*2 _are two primer pairs of PCR-CTPP. *F*_*s*1 _vs. F_*e*1 _and *R*_*s*1 _*vs*. *R*_*e*1 _indicate the start index *vs. *the end index of *P*_*f*1 _and *P_r1 _*in *T_D_*, respectively. *F*_*s*2 _*vs. F*_*e*2 _and *R*_*s*2 _*vs*. *R*_*e*2 _indicate the start index *vs. *the end index of *P*_*f*2 _and *P_r2 _*in *T_D_*, respectively. ${\bar{B}}_{i}$ is the complementary nucleotide of *B_i_*, which is described in formula (1). For example, if *B_i _*= 'A', then ${\bar{B}}_{i}$ = **'**T'; if *B_i _*= 'C', then ${\bar{B}}_{i}$ = **'**G', and *vice versa*.

The target SNP site is defined at the end positions of *P*_*f*2 _and *P*_*r*1_, which are indicated by the symbols *F*_*e*2 _and *R*_*e*1_, respectively. As described in Figure 1, a vector (*v*) with *F*_*l*1_, *P*_*l*1_, *R*_*l*1_, *F*_*l*2_, *P*_*l*2 _and *R*_*l*2 _is essential to design the CTPP primer sets. This vector is defined as follows:

(6) $$P_{v}=\left(F_{l}{}_{\text{1}},P_{l}{}_{\text{1}},R_{l}{}_{\text{1}},F_{l}{}_{\text{2}},P_{l}{}_{\text{2}},R_{l}{}_{\text{2}}\right)$$

*F*_*l*1_, *P*_*l*1_, *R*_*l*1_, *F*_*l*2_, *P*_*l*2 _and *R*_*l*2 _represent the lengths of forward primer 1, PCR product between *P*_*f*1 _and *P_r1_*, reverse primer 1, forward primer 2, PCR product between *P*_*f*2 _and *P_r2 _*and reverse primer 2, respectively. Consequently, the forward and reverse primers can be acquired from *P_v_*, which is the prototype of a chromosome in GA and is used to perform evolutionary computations as described in the following sections.

### Definition of the fitness function

The regular primer design constraints are used as values to design a fitness function to minimize the fitness value. The fitness function is defined as follows:

(7) $$\begin{array}{c} Fitness(P_{v})=\text{3}*(Len_{diff}(P_{v})+GC_{proportion}(P_{v})\\ +\;GC_{clamp}(P_{v}))+\text{1}0*(dimer(P_{v})\\ +\;hairpin\left(P_{v}\right)+specificity\left(P_{v}\right))\\ +\text{ 5}0*(T\text{m}(P_{v})\;+T{\text{m}}_{diff}(P_{v}))\\ +\text{ 1}00*vg\_T{\text{m}}_{diff}(P_{v})\\ +\text{ 6}0*PCR\text{len}\left(P_{v}\right) \end{array}$$

The weights (3, 10, 50, 60 and 100) of the fitness function are applied to estimate the importance of the primer constraints. These weights are set according to the experiential conditions for PCR-CTPP. They also accept adjustment based on the user experimental requirements.

#### Primer length

The feasible primer length for a PCR experiment is set between 16 and 28 bp. For longer primers, the *T*_m _is increased whereas the *T*_m _of relatively short primers is decreased. Accordingly, primers which are neither too long nor too short are suitable. We have limited the random values of *F*_*l*1_, *R*_*l*1_, *F*_*l*2 _and *R*_*l*2 _in an appropriate range; therefore, the primer length estimation is not considered to be joined to the fitness function.

A length difference (*Len_diff_*) of less than or equal to 3 bp between the *F*_*l*1_/*R*_*l*1_, *F*_*l*2_/*R*_*l*2_, and *F*_*l*1_/*R*_*l*2 _primer sets is considered optimal. The primer length difference function is defined as follows:

(8) $$Lendiff(Pv)=\left\{ \begin{array}{l} defect\_value=3\\ \text{if ABS (}Fl1-Rl1\text{)}\leq \text{3, }\\ \text{ then }defect\_value-1\\ \text{if ABS (}Fl2-Rl2\text{) }\leq \text{3, }\\ \text{ then }defect\_value-1\\ \text{if ABS (}Fl1-Rl2\text{)}\leq \text{3, }\\ \text{ then }defect\_value-1\\ \text{return }defect\_value \end{array}\right.$$

where *Len_diff_*(*P_v_*) has a maximal fitness value of 3; the fitness value is decreased when the length difference between a primer pair is less than or equal to 3 bp. ABS represents the absolute value.

#### GC content and GC clamp

The function *GC*%(*P*) is proposed to represent the ratio of G and C nucleotides appearing in a primer:

(9) $$GC\%(P)=\frac{Gnumber(P)+Cnumber(P)}{\left|P\right|}$$

where *P *represents a primer and | *P *| represents the length of primer *P*; *G_number_*(*P*) and *C_number_*(*P*) represent the numbers of the nucleotides G and C, respectively.

In general primer design, the typical GC proportion constraint is set between 40% and 60%. However, the designed CTPP primers contain the target SNP to limit the range of the GC proportion. To relax this constraint, the constraint of GC proportion in a primer is adjusted to between 20% and 80%. Function *GC_proportion_*(*P_v_*) is proposed with a maximal fitness value of 4 to lead the *GC*%(*P*) of CTPP primers corresponding to this constraint:

(10) $$GCproportion(Pv)=\left\{ \begin{array}{l} defect\_value=4\\ \text{if 20}\leq GC\%(Pf1)\leq 80,\;\\ \text{ then }defect\_value-1\\ \text{if 20}\leq GC\%(Pr\text{1})\leq 80,\;\\ \text{ then }defect\_value-1\\ \text{if 20}\leq GC\%(Pf2)\leq 80,\;\\ \text{ then }defect\_value-1\\ \text{if 20}\leq GC\%(Pr\text{2})\leq 80,\;\\ \text{ then }defect\_value-1\\ \text{return }defect\_value \end{array}\right.$$

To meet the presence of G or C nucleotides at the 3' terminal of a primer to ensure a tight localized hybridization bond, the function *GC_clamp_*(*P_v_*) is proposed with the maximal fitness value of 4 as follows:

(11) $$GCclamp(Pv)=\left\{ \begin{array}{l} defect\_value=4\\ \text{if 3' end of }Pf1\text{ is 'G' or 'C'},\\ \text{ then }defect\_value-1\\ \text{if 3' end of }Pr\text{1 is 'G' or 'C'},\\ \text{ then }defect\_value-1\\ \text{if 3' end of }Pf2\text{ is 'G' or 'C'},\\ \text{ then }defect\_value-1\\ \text{if 3' end of }Pr\text{2 is 'G' or 'C'},\\ \text{ then }defect\_value-1\\ \text{return }defect\_value \end{array}\right.$$

#### Melting temperature

The melting temperature (*T*_m_) for each CTPP primer must be considered carefully for PCR experiments. Here, we do not use the rough estimate 2 × (#A + #T) + 4 × (#G + #C), but a more elaborate equation containing the ionic strength, G and C content and length of the primer is concerned. The *T*_m _calculation formula for a primer is described as follows:

(12) $$\begin{array}{c} T{\text{m}}_{B}{}_{M}\left(P\right)=\text{81}.\text{5}+\text{16}.\text{6}*({\text{log}}_{\text{1}0}[{\text{Na}}^{+}])\\ +\;0.\text{41}*(\text{GC}\%)–\text{675}/\left|P\right| \end{array}$$

where *P *represents a primer and | *P *| represents the length of primer *P*; Na^+ ^is the molar salt concentration. The suffix BM represents the formula which was proposed by Bolton and McCarthy [23].

The function *T*m(*P_v_*) is proposed to confine a CTPP primer set ranging from 45°C and 62°C with the maximal fitness value of 4:

(13) $$T\text{m}(Pv)=\left\{ \begin{array}{l} defect\_value=4\\ \text{if 45}\leq T{\text{m}}_{BM}\text{(}Pf1)\;\leq 62,\\ \text{ then }defect\_value-1\\ \text{if 45}\leq T{\text{m}}_{BM}\text{(}Pr\text{1})\;\leq 62,\;\\ \text{ then }defect\_value-1\\ \text{if 45}\leq T{\text{m}}_{BM}\text{(}Pf2)\;\leq 62,\;\\ \text{ then }defect\_value-1\\ \text{if 45}\leq T{\text{m}}_{BM}\text{(}Pr\text{2})\;\leq 62,\;\\ \text{ then }defect\_value-1\\ \text{return }defect\_value \end{array}\right.$$

Similar *T*_m _between a primer pair is important to perform experiment in the same tube. The function *T*m*_diff_*(*P_v_*) is proposed with the maximal fitness value of 3 to guide the difference of the melting temperatures to less than or equal to 1°C:

(14) $$T\text{m}diff(Pv)=\left\{ \begin{array}{l} defect\_value=3\\ \text{if ABS }(T{\text{m}}_{BM}\text{(}Pf1)-T{\text{m}}_{B}\text{(}Pr\text{1}))\leq 1,\\ \text{ then }defect\_value-1\\ \text{if ABS }(T{\text{m}}_{BM}\text{(}Pf2)-T{\text{m}}_{B}\text{(}Pr\text{2}))\leq 1,\\ \text{ then }defect\_value-1\\ \text{if ABS }(T{\text{m}}_{BM}\text{(}Pf1)-T{\text{m}}_{B}\text{(}Pr\text{2}))\leq 1,\\ \text{ then }defect\_value-1\\ \text{return }defect\_value \end{array}\right.$$

In order to balance the *T*_m _values among a CTPP primers, function *Avg_T*m*_diff_*(*P_v_*) is proposed to calculate the average *T*_m _difference:

(15) $$\begin{array}{l} Avg\_T\text{m}diff(Pv)=[\text{ABS}(T{\text{m}}_{BM}\text{(}Pf1)-T{\text{m}}_{BM}\text{(}Pr\text{1}))\\ \;+\text{ ABS}(T{\text{m}}_{BM}\text{(}Pf2)-T{\text{m}}_{BM}\text{(}Pr\text{2}))\\ \;+\text{ABS}(T{\text{m}}_{BM}\text{(}Pf1)-T{\text{m}}_{BM}\text{(}Pr\text{2}))]/3 \end{array}$$

#### Dimer and hairpin

Primer dimers (annealing of two primers), such as cross-dimers (a forward primer and a reverse primer anneal to each other) and self-dimers (two forward primers or two reverse primers anneal to each other) must also be avoided. To check for the occurrence of primer dimers, the function *dimer*(*P_v_*) is proposed with the maximal fitness value of 10:

(16) $$dimer(Pv)=\left\{ \begin{array}{l} defect\_value\;=\;10\\ \text{if(}Pf1\text{ and }Pr\text{1) do not form a cross -dimer,}\\ \text{ then }defect\_value-1\\ \text{if(}Pf2\text{ and }Pr\text{2) do not form a cross -dimer,}\\ \text{ then }defect\_value-1\\ \text{if(}Pf2\text{ and }Pr\text{1) do not form a cross -dimer,}\\ \text{ then }defect\_value-1\\ \text{if(}Pf1\text{ and }Pr\text{2) do not form a cross-dimer,}\\ \text{ then }defect\_value-1\\ \text{if(}Pf1\text{ and }Pf\text{2) do not form a cross-dimer,}\\ \text{ then }defect\_value-1\\ \text{if(}Pr1\text{ and }Pr\text{1) do not form a cross-dimer,}\\ \text{ then }defect\_value-1\\ \text{if(}Pf1\text{ and }Pf\text{1) do not form a self-dimer,}\\ \text{ then }defect\_value-1\\ \text{if(}Pr1\text{ and }Pr\text{1) do not form a self-dimer,}\\ \text{ then }defect\_value-1\\ \text{if(}Pf2\text{ and }Pf\text{2) do not form a self-dimer,}\\ \text{ then }defect\_value-1\\ \text{if(}Pr2\text{ and }Pr\text{2) do not form a self-dimer,}\\ \text{ then }defect\_value-1\\ \text{return }defect\_value \end{array}\right.$$

The hairpin check is also implemented to avoid annealing due to the secondary structure of a primer. To check for the presence of a hairpin structure in CTPP primers, the function *hairpin*(*P_v_*) is proposed with the maximal fitness value of 4 as follows:

(17) $$hairpin(Pv)=\left\{ \begin{array}{l} defect\_value=4\\ \text{if }Pf1\text{ do not form a hairpin,}\\ \text{ then }defect\_value-1\\ \text{if }Pr\text{1 do not form a hairpin,}\\ \text{ then }defect\_value-1\\ \text{if }Pf2\text{ do not form a hairpin,}\\ \text{ then }defect\_value-1\\ \text{if }Pr\text{2 do not form a hairpin,}\\ \text{ then }defect\_value-1\\ \text{return }defect\_value \end{array}\right.$$

#### Specificity

Subsequently, the function *specificity*(*P_v_*) is proposed to check for repetition of each CTPP primer in the template DNA sequence to ensure its specificity. The PCR experiment may fail when a designed primer is not sequence-specific (i.e. it reappears in the genome). The fitness value of the primers (*P*_*f*1_, *P*_*r*1_, *P*_*f*2 _or *P*_*r*2_) appearing in *T_D _*is evaluated using a binary value, i.e., when the primers repeatedly appear in *T_D_*, the *specificity*(*P_v_*) is defined as 1; or else the *specificity*(*P_v_*) is defined as 0.

#### PCR product length

Finally, the function *PCR*len(*P_v_*) is proposed with the maximal fitness value of 7 to calculate the appropriate lengths of the PCR products. Three ratios - i.e. ratio1, ratio2 and ratio3 - are introduced to the function *PCR*len(*P_v_*) representing *P*_*l*1_, *P*_*l*2 _and *P*_*l*3_, respectively. The minimum length of PCR products needs to be greater than 100 bp.

(18) $$PCR\text{len}(Pv)=\left\{ \begin{array}{l} defect\_value=7\\ \text{if }Pl1\;>\text{ 100 , then }defect\_value-1\\ \text{if }Pl2\;>\text{ 100 , then }defect\_value-1\\ \text{if }Pl3\;>\text{ 100, then }defect\_value-1\\ \text{if }Pl1\text{ corresponds ratio1,}\\ \text{ then }defect\_value-1\\ \text{if }Pl2\text{ corresponds ratio2,}\\ \text{ then }defect\_value-1\\ \text{if }Pl3\text{ corresponds ratio3,}\\ \text{ then }defect\_value-1\\ \text{if all }Pl1\text{ and }Pl2\text{ and }Pl3\text{ correspond their ratios,}\\ \text{ then }defect\_value-1\\ \text{return }defect\_value \end{array}\right.$$

### Algorithm

**Figure 2:**
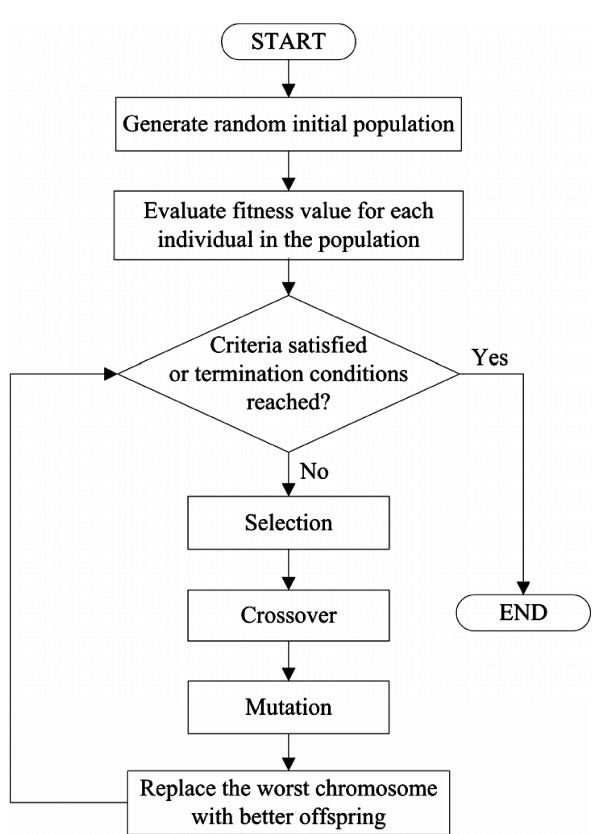
**Flowchart of the GA-based CTPP primer design**. At first, a random initial population is generated and then all fitness values of all chromosomes in the population are calculated by the fitness function. A judgment on termination conditions is carried out, and if the termination conditions are reached then the algorithm will be finished, or else the algorithm proceeds with the following processes. Selection, crossover and mutation operations are performed and finally the worst chromosomes are replaced by the better chromosomes. The procedure is repeated in the next iteration until the termination conditions are reached.

The proposed algorithm consists of five processes: (1) random initial population, (2) fitness evaluation, (3) selection, crossover, and mutation, (4) replacement, and (5) judgment on termination conditions. Figure 2 shows the flowchart of GA-based CTPP primer design. The five processes are described below:

#### (1) Random initial population

To start the algorithm, chromosomes *P_v _*= (F_*l*1_, *P*_*l*1_, *R*_*l*1_, *F*_*l*2_, *P*_*l*2_, *R*_*l*2_) of particular number are randomly generated for an initial population without duplicates. *F*_*l*1_, *R*_*l*1_, *F*_*l*2 _and *R*_*l*2 _are randomly generated between the minimum and the maximum of the primer length constraint. The minimum and maximum lengths of the primer length constraint are set to 16 and 28 bp, respectively. The PCR product lengths, *P*_*l*1 _and *P*_*l*2 _are randomly generated between 100 bp and *δ*_1_, and between 100 bp and *δ*_2_, respectively. (*δ*_1 _and *δ*_2 _are the maximum tolerant PCR product lengths of *P*_*l*1 _and *P*_*l*2 _shown in Figure 1)

#### (2) Fitness evaluation

The fitness value in the fitness function is used to ascertain that an individual chromosome is either good or bad. We use formula (7) to evaluate the fitness values of all chromosomes in the population for related chromosomal operations later.

#### (3) Selection, crossover, and mutation

**Figure 3:**
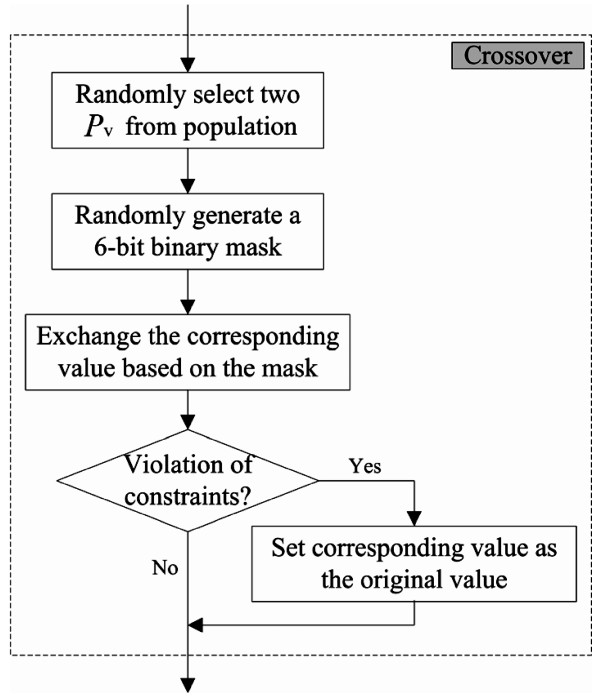
**Crossover flowchart for CTPP primer design**. Two *P_v _*from the population are randomly selected for crossover. At first, a six bit binary mask is generated and indicates which variables need to be exchanged. All exchanged variables are checked for violation of a constraint. If a constriction is violated, the exchanged variables will be restored, or else the process goes on to the next step.

**Figure 4:**
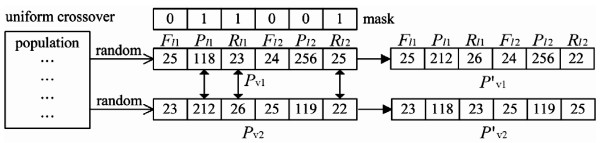
**An example of a crossover operation for CTPP primer design**. First, randomly select two *P_v _*from the population, for example *P*_*v*1 _= (25, 118, 23, 24, 256, 25) and *P*_*v*2 _= (23, 212, 26, 25, 119, 22). Then randomly generate a mask of 6 binary bits i.e., 011001, and based on this mask, exchange the value of *P*_*l*1_, *R*_*l*1 _and *R*_*l*2_. Finally, the new offsprings *P*'_*v*1 _= (25, 212, 26, 24, 256, 22) and *P*'_*v*2 _= (23, 118, 23, 25, 119, 25) are generated.

**Figure 5:**
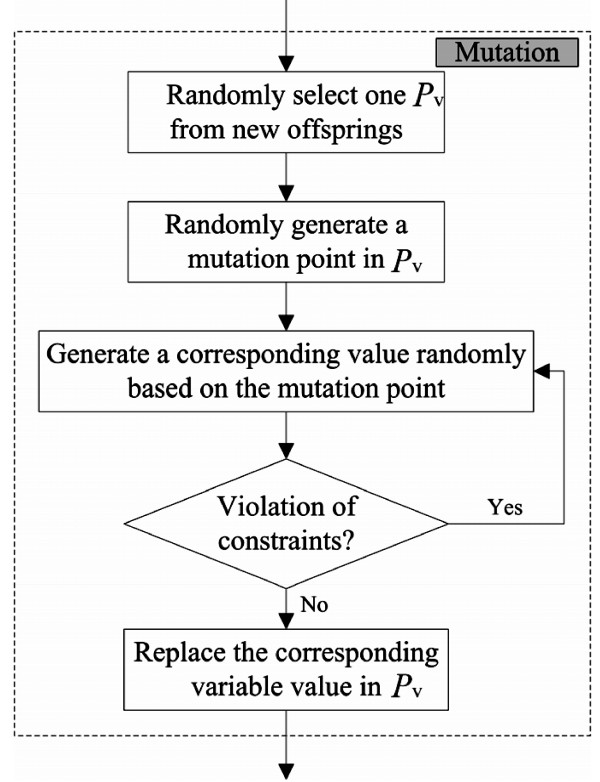
**Mutation flowchart for CTPP primer design**. When a mutation operation is performed, a mutation point in *P_v _*will be randomly selected and a corresponding value generated. Then the variable will be checked for violation of a constriction. If a constriction is violated, a random point is reselected and the variable is regenerated; otherwise the original value is replaced by the variable and the process continues in the next step.

**Figure 6:**
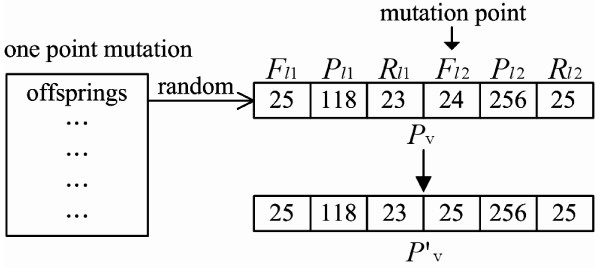
**An example of a mutation operation for CTPP primer design**. At the beginning, a *P_v _*= (25, 118, 23, 24, 256, 25) is randomly selected from the offsprings. Then one mutation point of the position *F*_*l*1_, *P*_*l*1_, *R*_*l*1_, *F*_*l*2_, *P*_*l*2 _or *R*_*l*2 _is randomly generated. In our example the position the mutation point is *F*_*l*2_. Then a random value between 16 and 28 of *F*_*l*2 _is generated. This value replaces the corresponding variable value in *P_v_*. Finally, the new offspring *P*'*_v _*= (25, 118, 23, 25, 256, 25) is generated.

In GA, the processes for evolutionary computation include selection, crossover and mutation. Here, random selection is applied to select two chromosomes from the population. The two selected chromosomes are processed by the crossover operation. Uniform crossover is used to implement the crossover operation. The flowchart of the crossover process is shown in Figure 3, and an example of the crossover operation is shown in Figure 4. One-point mutation is applied in the proposed GA. The mutation process flowchart is shown in Figure 5, and an example of the mutation operation is shown in Figure 6.

#### (4) Replacement

After the evolutionary computation processes have been implemented, the two worst chromosomes in a population are replaced by the new offsprings, and the process is repeated in the next generation.

#### (5) Judgment on termination conditions

Once an optimal solution of chromosomes (i.e. the fitness value is 0) or the maximum number of iterations is achieved, the GA is terminated.

### Other important criteria for CTPP primer design

There is already one alternative nucleotide in the defined SNP for the CTPP primers *P*_*f*2 _and *P*_*r*1_. If a further SNP exists in any other CTPP primers, such as *P*_*f*1 _and *P*_*r*2_, the *T*_m _between all primers is more dynamic and is difficult to optimize. Accordingly, we avoid designing primers *P*_*f*1 _and *P*_*r*2 _with extra SNPs, i.e., all the designed primers for *P*_*f*1 _and *P*_*r*2 _including SNPs are eliminated without further processing.

## Results

### Dry dock experiments

#### The environment

The proposed algorithm was run using Xeon(TM) CPU 3.20 GHz × 2 and 2 GB RAM under the Microsoft Windows XP SP2 and JAVA 5.0 platforms.

#### Parameter settings

Four main parameters are set for the proposed algorithm, i.e. the number of iterations (generations), the population size, the probability of crossover and the probability of mutation. The respective values were 1000, 50, 0.6 and 0.001; the values are based on DeJong and Spears' parameter settings [24]. The population size was then set at 1000 and the other parameters were held constant; only the population size was increased (see Discussion for detail). The PCR product length was set to three ratios (ratios 1, 2, and 3) at 8, 13, and 20, respectively, which allowed for the distinct separation of PCR bands via gel electrophoresis. These ratios were chosen based on our previously conducted PCR experiments.

#### Results for GA-based CTPP primer design in the example of the SLC6A4 gene

A point mutation in the SLC6A4 gene was recently identified and shown to be associated with autism spectrum disorders [25], psychosis [26], and bipolar [27] patients. The SLC6A4 gene is the member 4 for a solute carrier family 6 (neurotransmitter transporter, serotonin). The common constraints for CTPP primer design were used, including a flanking length of 500 bp, primer length of between 16~28 bp, GC% between 20~80%, primer *T*_m _between 45 and 62°C, difference of CTPP primer *T*_m _of less than 1°C, product length larger than 100 bp, and clearly separated PCR bands in gel electrophoresis. The SNPs for SLC6A4 gene (288 SNPs) are used as an example in this study which excluded the deletion/insertion polymorphisms (DIPs) and multi-nucleotide polymorphisms (MNPs). These SNPs were retrieved with 500 bp flanking length (at both sides of SNP) from SNP-Flankplus (http://bio.kuas.edu.tw/snp-flankplus/) [28]; the reference cluster IDs of these SNPs are shown in http://bio.kuas.edu.tw/ga-ctpp/dataset.jsp.

The entire CTPP primer set results are provided at http://bio.kuas.edu.tw/ga-ctpp/appendix.jsp and statistics of the results based on the common constraints of GA-based CTPP primers are shown in Table 1. For the 288 SNPs, there are 1152 representative primers for GC%, GC clamp, *T*_m_, hairpin and specificity criteria (288 × 4 = 1152; a CTPP primer set contains four primers for a SNP). For the length difference, *T*_m _difference and the product length criteria, there are only 864 (288 × 3 = 864; a CTPP primer set which contains four primers to lead three product lengths). The number of dimer is 2880 (each primer may form self-dimer and two different primers may form cross-dimer in a CTPP primer set). The primer lengths are all between 16 and 28 bp. In ID1 as shown in Table 1, the parameter settings are based on DeJong and Spears, the designed primer length difference violated the parameter settings for 215 primers (215/864). Most of the primer length differences were between 0 and 5 bp (data not shown). For GC%, 30 primers were less than 20%, 25 primers were more than 80% and the GC% distribution was mainly between 30% and 70% (data not shown). Approximately half of the primers (645/1152) did not satisfy the GC clamp criteria. Most of the designed primers also satisfied *T*_m _(998/1152); however, only approximately 23.6% (204/864) of the primer pairs satisfied the *T*_m _difference criteria. The criteria for product length were satisfied in approximately 71.2% (615/864) of the designed primer pairs. For the criteria for primer dimer, hairpin and specificity, few primers were problematic (128/2880, 162/1152 and 35/1152, respectively).

**Table 1:** The statistics of the designed CTPP primers showing how many primers satisfied the common constraints for SNPs of the SLC6A4 gene*

						Constraints				
**ID**		**primer** **length difference**	**GC%**	**GC clamp**	***T***_**m**_	***T***_**m **_**difference**	**product length**	**dimer**	**hairpin**	**specificity**

1	Results	649/864	1107/1152	645/1152	998/1152	204/864	615/864	2752/2880	990/1152	1117/1152
2		684/864	1115/1152	653/1152	1103/1152	512/864	608/864	2760/2880	969/1152	1121/1152

* The results of ID 1 and ID2 are the parameter settings based on DeJong and Spears but ID2 is modified with an increased population size to 1000.

### Genotyping experiment

#### Materials

One SNP rs12449783 in the SLC6A4 gene was taken as an example for the genotyping test. Three DNA samples with three known different SNP genotypes for rs12449783 were used to demonstrate the effectiveness of the GA-based CTPP primer design.

#### Validation of SNP genotyping by GA-based PCR-CTPP and TaqMan probe

The designed CTPP primer set for rs12449783 in the SLC6A4 gene is given in Table 2. DNA samples were added to the PCR reaction mixture (10 μl) containing 1 μl of 10× PCR buffer, 0.3 μl of 50 mM MgCl_2_, 0.2 μl of 10 mM dNTPs, 0.6 μl of DMSO, 0.14 μl of 5 U Platinum Taq enzyme (Invitogen corp.), 0.12 μl of 350 μg/ml primer mix (1:1), and 7.64 μl of DNA in water. Primer mixtures of several combinations were used: *P*_*f*1_/*P*_*r*1 _and *P*_*f*2_/*P*_*r*2_, and *P*_*f*1_/*P*_*r*2 _(Figure 1). The used PCR program had the following paramaters: 94°C (4 min); 49 cycles at 94°C for (30 s), 50°C for (20 s), 72°C (20 s); and 72°C (5 min). PCR products were separated by electrophoresis on a 1.5% regular agarose gel followed by ethidium bromide staining.

**Table 2:** The information for the designed CTPP primers for rs12449783 of the SLC6A4 gene

	CTPP primer set for rs12449783	Length (bp)	GC%	***T***_**m**_	***T***_**m-diff **_**(°C)**	Product size (bp)
*P*_*f*1_:	GATTATTAGTAGTTTCTGCA	20	30	50.96		
*P*_*r*1_:	TTCTTTATGAATACCAGAC**G**	20	35	51.37	*P*_*f*1_/*P*_*r*1_: 0.41	*P*_*f*1_/*P*_*r*1_: 228
*P*_*f*2_:	AGAAAGTTACAGACTAGCA**A**	20	30	51.37	*P*_*f*2_/*P*_*r*2_: 0.41	*P*_*f*2_/*P*_*r*2_: 105
*P*_*r*2_:	ATGTTTAATCTCTGAGAAGA	20	35	51.37	*P*_*f*1_/*P*_*r*2_: 0	*P*_*f*1_/*P*_*r*2_: 294

The bold font represents a SNP.

**Figure 7:**
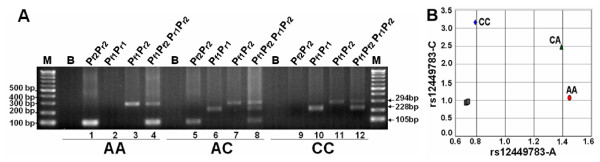
**The SNP genotyping results of rs12449783 by (A) PCR-CTPP running in gel electrophoresis and (B) by TaqMan probe**. (A) M and B indicate the 100 bp markers and blank, respectively. CC, AA, and AC indicate the control DNAs with known genotypes for CC, AA, and AC, respectively. The *P*_*f*2_/*P*_*r*2 _(lanes 1, 5, and 9) and *P*_*f*1_/*P*_*r*1 _(lanes 2, 6, and 10) primer pairs are designed to specifically amplify the A- and C-containing alleles for genotypes AA and AC *vs*. genotypes CC and AC, respectively. The *P*_*f*1_/*P*_*r*2 _(lanes 3, 7, and 11) primer pair is designed to amplify the A- or C-containing alleles (CC, AA, and AC). For PCR-CTPP using four primers in one tube, it is marked with "*P*_*f*1_, *P*_*f*2_, *P*_*r*1_, *P*_*r*2_" for lanes 4, 8, and 12. The relative positions for *P*_*f*1_, *P*_*f*2_, *P*_*r*1_, and *P*_*r*2 _are shown in Figure 1.

In the principle of PCR-CTPP, two paired primers (four primers; *P_f1_*, *P_r2_*, *P_r1_*, and *P_r2_*) should be placed in one tube. Accordingly, when it is succeeded, two DNA bands are amplified for the heterozygotes and three DNA bands for the heterozygotes. As shown in Figure 7A, the samples were performed in PCR-CTTP using four CTPP primers (*P_f1_*, *P_r2_*, *P_r1_*, and *P_r2_*) within one tube (lanes 4, 8, and 12). Moreover, we also performed these PCR reactions separately for each set of CTPP primers (*P_f2_P_r2 _*for lanes 1, 5, and 9; *P_f1_P_r1 _*for lanes 2, 6, and 10; and *P_f1_P_r2 _*for lanes 3, 7, and 11, respectively), to clearly validate the performance for each combination of the CTPP primers (*P_f2_P_r2_*; *P_f1_P_r1_*; or *P_f1_P_r2_*) designed by our proposed computational algorithm.

As in Figure 7A for either the four CTPP primers or three different sets of two combined CTPP primers, the samples with AA genotype showed AA-negative for 228-bp (*P_f1_P_r1_*) and AA-positive for 105- (*P_f2_P_r2_*) and 294-bp (*P_f1_P_r2_*); the samples with CC genotype showed CC-negative for 105-bp and CC-positive for 228- and 294-bp; and the samples with AC genotype showed AC-positive for 228-, 105- and 294-bp. Accordingly, the bands of the A- and C-alleles-specific PCR amplifications were successfully demonstrated for AA/AC (105-bp) and CC/AC (228-bp), respectively. The internal positive PCR controls for all alleles (i.e., A and C) were confirmed. Therefore, it is clearly demonstrated that our proposed algorithm is able to provide the validated primers for PCR-CTPP.

Using the same samples in Figure 7A, the CTPP results were examined further using the TaqMan probes which were ABI no. hcv_7911133, VIC-/FAM-labels for ACACATAGAAAGTTACAGACTAGCA[A/C]GTCTGGTATTCATAAAGAATTGTGA, respectively. The TaqMan probe program was performed by a 2 step-protocol built-in the ABI real-time system (50°C, 2 min (stage 1, 1 cycle), 95°C, 10 min (stage 2, 1 cycle), 95°C, 15 sec (stage 3, 40 cycles), and 60°C, 1 min.). As shown in Figure 7B, the samples with AA, AC, and CC genotypes for rs12449783 in PCR-CTPP results (Figure 7A) were confirmed to be the same using the TaqMan probe assay. Therefore, the primer information for PCR-CTPP designed by our proposed algorithm was well proved.

## Discussion

To date, many primer design approaches have been proposed, such as dynamic programming [29], parthenogenetic algorithm MG-PGA [30], greedy algorithm [31], heuristic algorithm [32], genetic algorithm [21,33], memetic algorithm [28] and any others. However, most of these methods do not provide the SNP genotyping function. In contrast, we reported the brief idea of the GA-CTPP method for primer design of SNP genotyping in the IEEE BIBE 2009 conference [34]. The differences between them and the improvements of the algorithm proposed in this study are described in the additional file 1. In this study, we present an improved GA-based algorithm which has been shown to be a robust search and optimization method for a number of practical problems, especially for highly complex problems for SNP genotyping with the CTPP primers design function. We had used electrophoresis to validate the reliability of the GA-based CTPP primer design method.

### Influence of annealing temperatures

In PCR-CTPP, the designed annealing temperatures of primers are more important than in PCR-RFLP. When the *T*_m _value is similar among the four primers of PCR-CTPP, the PCR competition between all possible DNA products is balanced [10]. When the annealing temperature is low, the PCR reactions are non-specific, leading to incorrect heterozygous genotyping. Therefore, a competitive or specific amplification is important to correctly genotype SNPs using PCR-CTPP. This problem is resolved by computationally finding similar *T*_m _values for the four CTPP primers and by experimentally adjusting the annealing temperature for the PCR [10,35]. The GA used in this study to design the PCR-CTPP primers improves the efficiency by finding almost identical *T*_m _values for the four primers. The *in silico *testing of the proposed GA-based PCR-CTPP primer design showed it to fit the *T*_m _constraint to the primers reliably (Table 1).

### Typical primer design constraints concerned

Since the *T*_m _is important to our proposed GA-based PCR-CTPP method, further basic research is required to determine other factors to improve this automated PCR-CTPP system. This study is also concerned with the typical primer design constraints, such as primer length, primer length difference, GC proportion, GC clamp, dimer of primer pair (including cross-dimers and self-dimers), hairpin, PCR product length and specificity etc. as described in the Methods section.

### Effect of population size

Dejong and Spears' parameter settings are the standard for most GAs, and for this reason, they were used in the present study. Typically, crossover is usually applied at more than or equal to the rate of 0.6, and the mutation rate is equal to 0.001 [24]. However, the population size 50 of Dejong and Spears's parameter settings is too small to provide the necessary sampling accuracy for the design of CTPP primer sets. Consequently, we tested the population size for 100, 200, 300, 400, 500, 600, 700, 800, 900, and 1000 to evaluate the primer design performance. When the population size was increased to 1000, it provides the more accurate sampling (as shown in the additional file 2). As shown in Table 1 ID2, the number of primers that satisfy the constraints was increased to 9.11% and 35.65% for the *T*_m _and the *T*_m _difference constraint, respectively. For other constraints, the numbers of satisfied constraints were almost similar. The results demonstrate that the increased population size can aid in the search for more feasible CTPP primer sets.

### Available for GA-CTPP

The GA-CTPP can be accessed at http://bio.kuas.edu.tw/ga-ctpp/. GA-CTPP designs appropriate two-pair primers to genotype a defined SNP based on the parameter settings of DeJong and Spears. Parameter settings or the primer design conditions can be changed individually by users based on their requirements. When the input sequence contains multiple SNPs, the first SNP will be taken as the defined SNP to design CTPP primer sets. GA-CTPP reports an optimal set of confronting two-pair primers through a text file that records all information of the CTPP primer set for genotyping of the defined SNP.

## Conclusions

PCR-CTPP may replace PCR-RFLP because the restriction enzyme digestion step can be omitted, resulting in lower costs and shorter genotyping times [10]; however, the PCR-CTPP is less developed for its computational tool providing PCR-CTPP primer design. A novel strategy for PCR-CTPP primer design has been introduced in this paper and the freely available web server implemented with this method was also constructed. With experimental validation, our proposed GA-based method is a useful algorithm to design feasible CTPP primers and it conforms to most of the PCR-CTPP constraints.

## Availability and requirements

Project name: GA-CTPP: Confronting two-pair primer design using genetic algorithm.

Project home page: http://bio.kuas.edu.tw/ga-ctpp/

Operating system(s): Operating systems free with web browser.

Programming language: Java

Other requirements: Java 1.5

License: none for academic users. For any restrictions regarding the use by non-academics please contact the corresponding author.

## Authors' contributions

C-HY coordinated and oversaw this study. Y-HC participated in the design of the algorithm, and wrote the program and the manuscript. L-YC provided the biochemistry background and introduced the bioinformatics needed for primer design. H-WC performed and verified the PCR experiment, and modified the manuscript. All authors read and approved the final manuscript.

## Supplementary Material

Additional file 1 **'The differences between our previous publication in BIBE 2009 conference **[34]**and this study'**. Click here for file

Additional file 2 **'The performances for primer design using our proposed GA-CTPP algorithm between different population sizes of Dejong and Spears's parameter settings'**. Click here for file
