# Supplementary material for: Confronting two-pair primer design for enzyme-free SNP genotyping based on a genetic algorithm
Source: BMC Bioinformatics. 2010 Oct 13;11:509. doi: 10.1186/1471-2105-11-509 (PMC2964683; doi:10.1186/1471-2105-11-509)
Supplement: Additional file 1 — 'The differences between our previous publication in BIBE 2009 conference [34]and this study'. [file 1471-2105-11-509-S1.DOC]

**Additional file 1**

**The differences for previous publication in IEEE BIBE 2009 conference**

In the past, we had published the brief version of the GA-CTPP method in this conference proceeding. However, this paper published in the BMC Bioinformatics performs more detail works which are different from the work published in the IEEE BIBE 2009 conference proceeding [1].

The conference is a way for research communication. We proposed our method and discussed with peers in the IEEE BIBE 2009 conference. Some ideas and problems were collected at this conference. It inspired us to improve the GA-based primer design method. In order to promote the quality of the study in our proposed method, we performed some new works different from the original conference paper. The major differences are described below in detail.

1. ***Different data set***

In original conference paper, only thirty SNPs of the Janus kinase 2 gene were tested for dry dock experiments. However, in the manuscript, the SLC6A4 gene with 288 SNPs were tested for dry dock experiments. The test ratio is 1: 9.6.

1. ***About fitness function evaluate***

In original conference paper, the definition of the fitness function only shows the formula of fitness function. It lacks the complete descriptions for primer constraint functions. However, the manuscript describes the entire primer constraint functions in detail. The evaluate primer constraint functions in the manuscript is more precise.

For examples:

*i) For the length difference constraint function*

The original conference paper describes the *Len_diff_*(*P_v_*) is used to check whether the length difference of a primer pair exceeds 3 bps. A length difference of 3 bps for the forward primer 1 and the reverse primer 1, the forward primer 2 and the reverse primer 2, and the forward primer 2 and the reverse primer 1 is considered to be optimal.

The manuscript describes it as a length difference (*Len_diff_*) less than or equal to 3 bp between the *F_l_*_1_/*R_l_*_1_, *F_l_*_2_/*R_l_*_2_, and *F_l_*_1_/*R_l_*_2_ primer sets is considered optimal. The primer length difference function is defined as follows:

|  | (8) |
| --- | --- |

where *Len_diff_*(*P_v_*) has a maximal fitness value of 3; the fitness value is decreased when the length difference between a primer pair is less than or equal to 3 bp. ABS represents the absolute value.

*ii) For the GC content and GC clamp functions*

The original conference paper describes the function for the GC proportion in a primer is denoted *GC*%(*P*), a value that calculates the ratio of nucleotides of G and C that appear in a primer. The appropriate GC proportion in a primer should be between 40% and 60%. However, CTPP primers for specific SNP site limit the range of GC proportion. In order to relax the constraint, in the study, we adjust the constraint of GC proportion in a primer between 20% and 80%. *GC_proportion_*(*P_v_*) function is used to check whether the *GC*%(*P*) of CTPP primers corresponding the constraint. And the function *GC_clamp_*(*P_v_*) is used to check whether the 3’ terminal end of a primer is G or C.

The manuscript describes them as:

The function *GC*%(*P*) is proposed to represent the ratio of G and C nucleotide appearing in a primer:

|  | (9) |
| --- | --- |

where *G_number_*(*P*) and *C_number_*(*P*) represent the numbers of the nucleotide G and C, respectively.

In general primer design, the typical GC proportion constraint is set between 40% and 60%. However, the designed CTPP primers contain the target SNP to limit the range of the GC proportion. To relax this constraint, the constraint of GC proportion in a primer is adjusted to between 20% and 80%. Function *GC_proportion_*(*P_v_*) is proposed with a maximal fitness value of 4 to lead the *GC*%(*P*) of CTPP primers corresponding this constraint:

|  | (10) |
| --- | --- |

To meet the presence of G or C nucleotide at the 3’ terminal of a primer to ensure a tight localized hybridization bond, the function *GC_clamp_*(*P_v_*) is proposed with the maximal fitness value of 4 as follows:

|  | (11) |
| --- | --- |

*iii) For the melting temperature related functions*

The original conference paper describes the melting temperature (*T*_m_) of a primer is calculated by a formula that was proposed by Bolton and McCarthy [2]. Function *T*m(*P_v_*) is used to check whether the melting temperatures of a CTPP primer set are between 45^o^C and 62^o^C, and function *T*m*_diff_*(*P_v_*) is used to check whether the difference of the melting temperatures exceeds 1^o^C. In order to balance *T*_m_ between primers, function *Avg_T*m*_diff_*(*P_v_*) is employed to calculate the average *T*_m_ difference between two-pair primers.

The manuscript with more complete descriptions about them:

The melting temperature (*T*_m_) for each CTPP primer must be considered carefully for PCR experiments. Here, we do not use the rough estimate 2 × (#A + #T) + 4 × (#G + #C), but a more elaborate equation takes into the ionic strength, G and C content and length of the primer. The *T*_m_ calculation formula for a primer is described as follows:

| *T*m*_BM_*(*P*) = 81.5 + 16.6 * (log_10_[Na^+^]) + 0.41 * (GC%) – 675 / | *P* | | (12) |
| --- | --- |

where *P* represents a primer and | *P* | represents the length of primer *P*; Na^+^ is the molar salt concentration. The suffix BM represents the formula which was proposed by Bolton and McCarthy [2].

The function *T*m(*P_v_*) is proposed to confined a CTPP primer set ranging from 45^o^C and 62^o^C with the maximal fitness value of 4:

|  | (13) |
| --- | --- |

Similar *T*_m_ between a primer pair is important to experiment in a tube. The function *T*m*_diff_*(*P_v_*) is proposed with the maximal fitness value of 3 to guide the difference of the melting temperatures to less than or equal to 1^o^C:

|  | (14) |
| --- | --- |

In order to balance the *T*_m_ values among a CTPP primers, function *Avg_T*m*_diff_*(*P_v_*) is proposed to calculate the average *T*_m_ difference:

|  | (15) |
| --- | --- |

*iv) For the dimer and hairpin functions*

The original conference paper describes them as:

Annealing between two primers (called a dimer) is suggested to be avoided. The annealing including a forward primer and a reverse primer (cross-dimers); a forward and a forward primer, and a reverse and a reverse primer (self-dimers). The function *dimer*(*P_v_*) is used to check whether primers anneal to each other. And the hairpin check is also important. A primer should avoid being annealed by itself or else it will form a hairpin. The function $\text{hairpin}(Pv)$ is used to check for this condition in a CTPP primer set.

The manuscript describes them as:

Primer dimers (annealing of two primers), such as cross-dimers (a forward primer and a reverse primer anneal to each other) and self-dimers (two forward primers or two reverse primers anneal to each other) must also be avoided. To check for the occurrence of primer dimers, the function *dimer*(*P_v_*) is proposed with the maximal fitness value of 10:

|  | (16) |
| --- | --- |

The hairpin check is also implemented to avoid annealing due to the secondary structure of a primer. To check for the presence of a hairpin structure in CTPP primers, the function *hairpin*(*P_v_*) is proposed with the maximal fitness value of 4 as follows:

|  | (17) |
| --- | --- |

*v) For the PCR product length function*

The original conference paper describes the function is employed to estimate appropriate PCR product length. Three ratios, ratio1, ratio2 and ratio3 are applied to the *PCR*len*_ratio_*(*P_v_*) function and represent *P_l_*_1_, *P_l_*_2_ and *P_l_*_3_, respectively. And the minimum product length must more than 100 bps.

The manuscript describes it as:

Finally, the function *PCR*len(*P_v_*) is proposed with the maximal fitness value of 7 to calculate the appropriate lengths of the PCR products. Three ratios, i.e. ratio1, ratio2 and ratio3, are introduced to the function *PCR*len(*P_v_*) representing *P_l_*_1_, *P_l_*_2_ and *P_l_*_3_, respectively. The minimum length of PCR products needs to be greater than 100 bp.

|  | (18) |
| --- | --- |

The manuscript characterizes the above functions for further providing the calculation for their respective fitness value.

1. ***The wet experiment validation***

The original conference paper lacks the wet experiment validation, while the manuscript provides the wet experiment validation for further considering its practical usability.

1. ***The parameter settings for proposed method***

The manuscript uses the standard parameter settings based on DeJong and Spears’ parameter settings [3], and increases the population size parameter to 1000 to improve the results of the dry dock experiments. It also discusses the effect of population size in the “Discussion” section. Nevertheless, the parameter settings in the original conference paper are not a standard one.

The parameter settings are listed below:

| **parameter settings** | **The original conference paper** | **The manuscript** | |
| --- | --- | --- | --- |
|  |  | **Parameters 1** | **Parameters 2** |
| the number of iterations (generations) | 100 | 50 | 1000 |
| the population size | 100 | 50 | 50 |
| the probability of crossover | 1.0 | 0.6 | 0.6 |
| the probability of mutation | 0.01 | 0.001 | 0.001 |

1. ***The flowchart and examples of the proposed method***

The manuscript provides the whole flowchart and examples of the proposed method, but the original conference paper does not.

For examples, the Figure 2~6 are the flowchart of the GA-based CTPP primer design, crossover flowchart for CTPP primer design, an example of a crossover operation for CTPP primer design, mutation flowchart for CTPP primer design, and an example of a mutation operation for CTPP primer design, respectively. They are shown as follows:


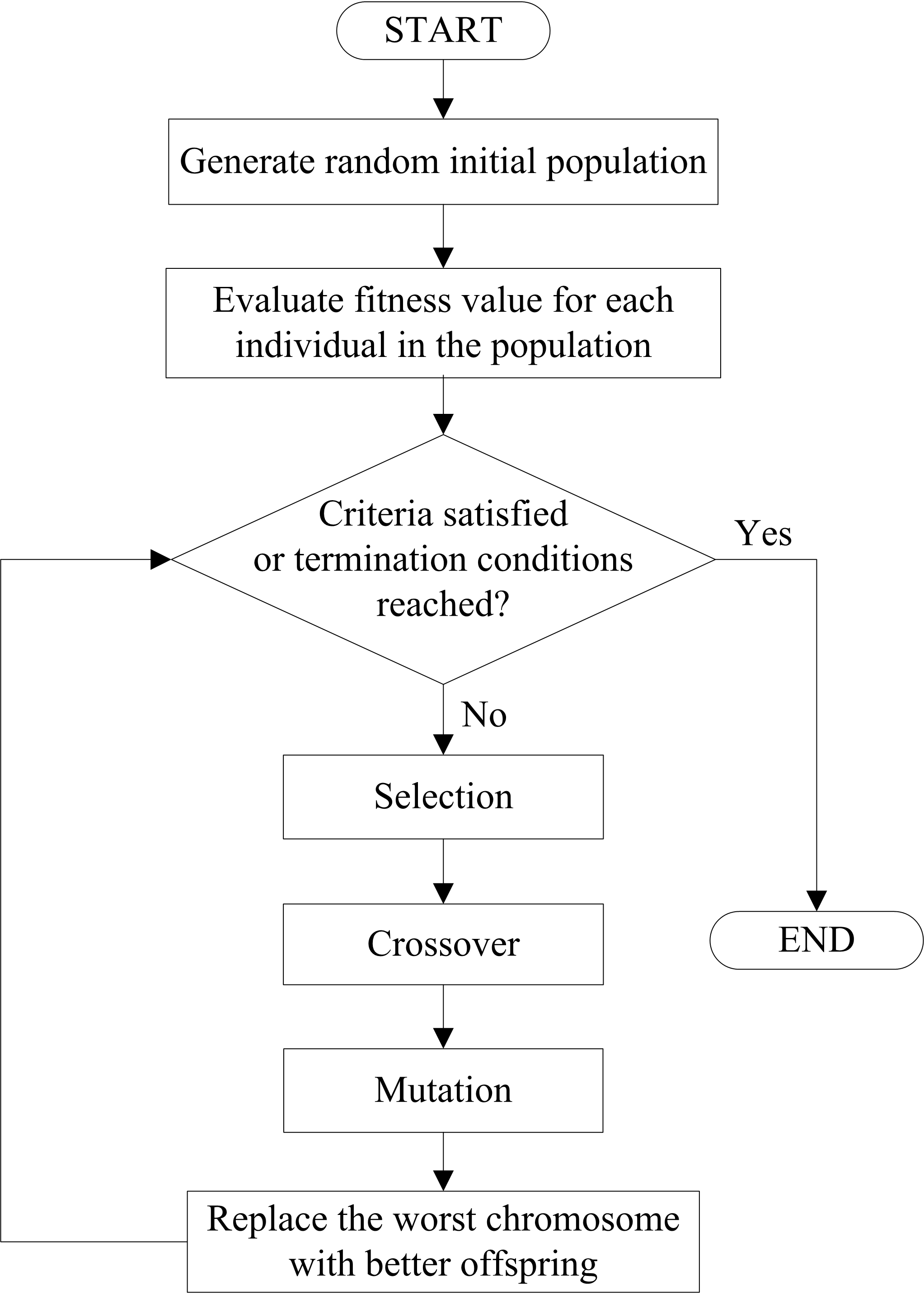


## Figure 2. Flowchart of the GA-based CTPP primer design. At first, a random initial population is generated and then all fitness values of all chromosomes in the population are calculated by the fitness function. A judgment on termination conditions is carried out, and if the termination conditions are reached then the algorithm will be finished, else the algorithm proceeds with the following processes. Selection, crossover and mutation operations are performed and finally the worst chromosomes are replaced by the better chromosomes. The procedure is repeated in the next iteration until the termination conditions are reached.


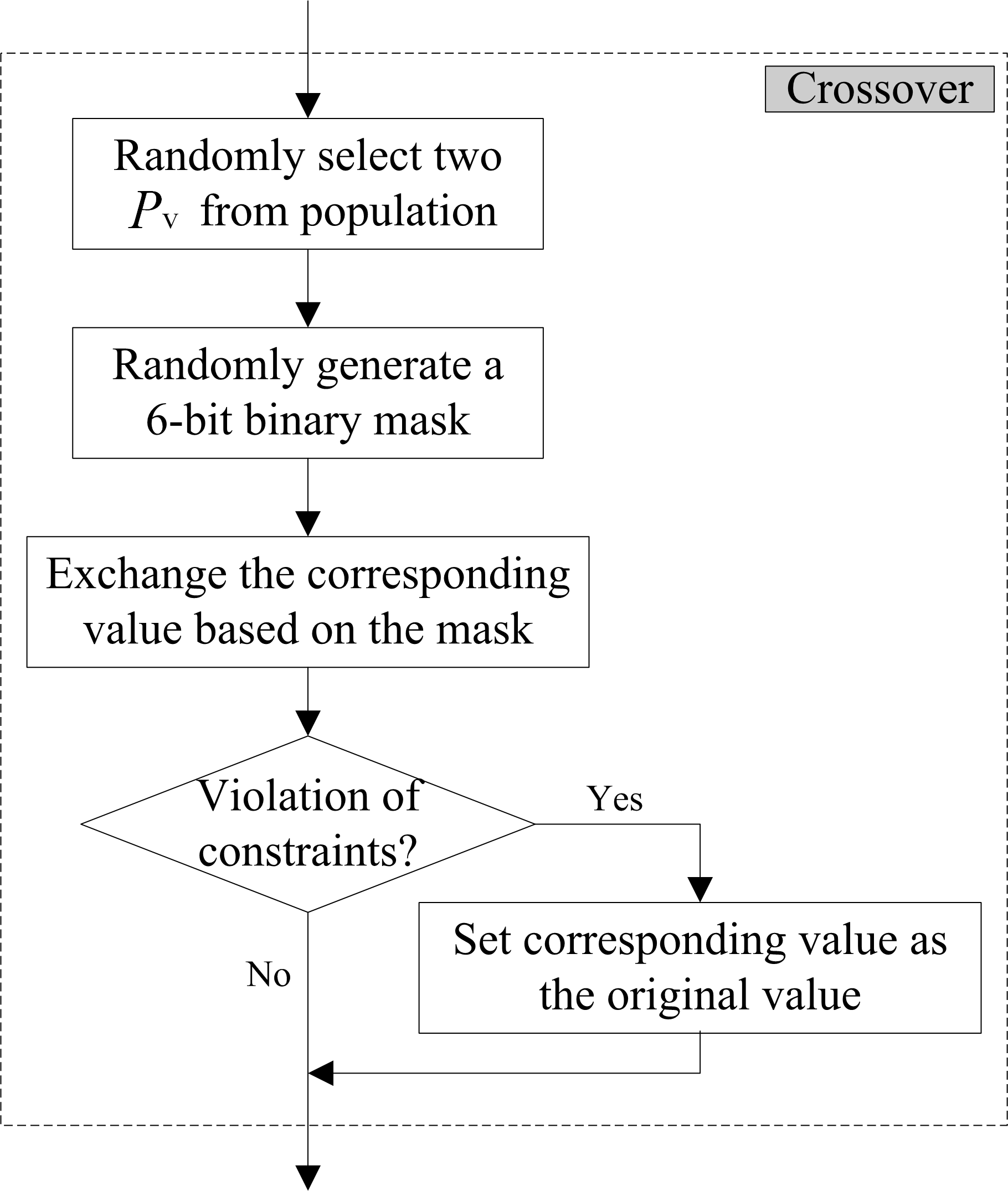


**Figure 3.** Crossover flowchart for CTPP primer design. Two *P_v_* from the population are randomly selected for crossover. At first, a six bit binary mask is generated and indicates which variables need to be exchanged. All exchanged variables are checked for violation of a constraint. If a constriction is violated, the exchanged variables will be restored, else the process proceeds to the next step.


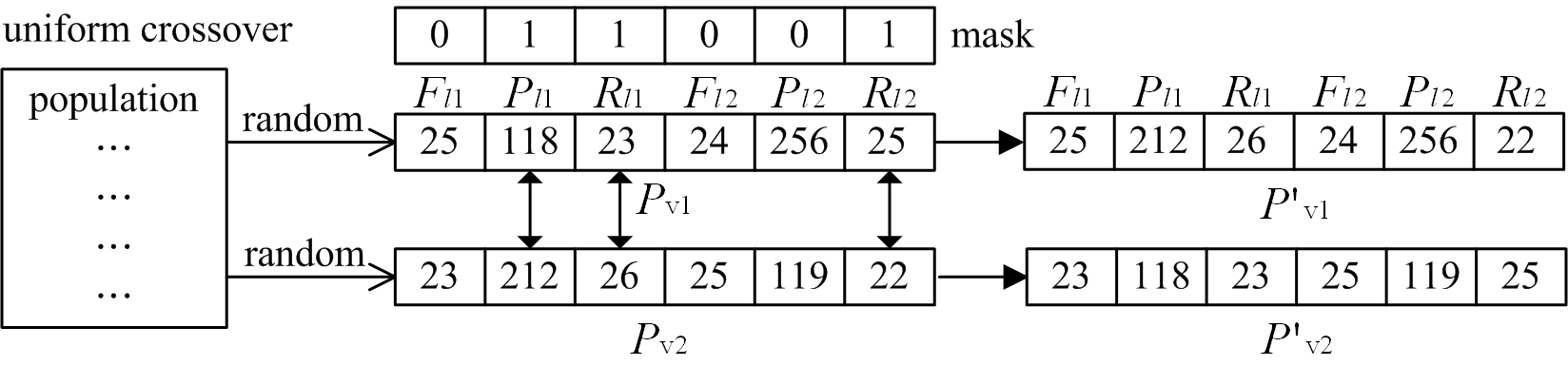


**Figure 4.** An example of a crossover operation for CTPP primer design. First, randomly select two *P_v_* from the population, for example *P_v_*_1_ = (25, 118, 23, 24, 256, 25) and *P_v_*_2_ = (23, 212, 26, 25, 119, 22). Then randomly generate a mask of 6 binary bits i.e., 011001, and based on this mask, exchange the value of *P_l_*_1_, *R_l_*_1_ and *R_l_*_2_. Finally, the new offsprings *P*'*_v_*_1_ = (25, 212, 26, 24, 256, 22) and *P*'*_v_*_2_ = (23, 118, 23, 25, 119, 25) are generated.


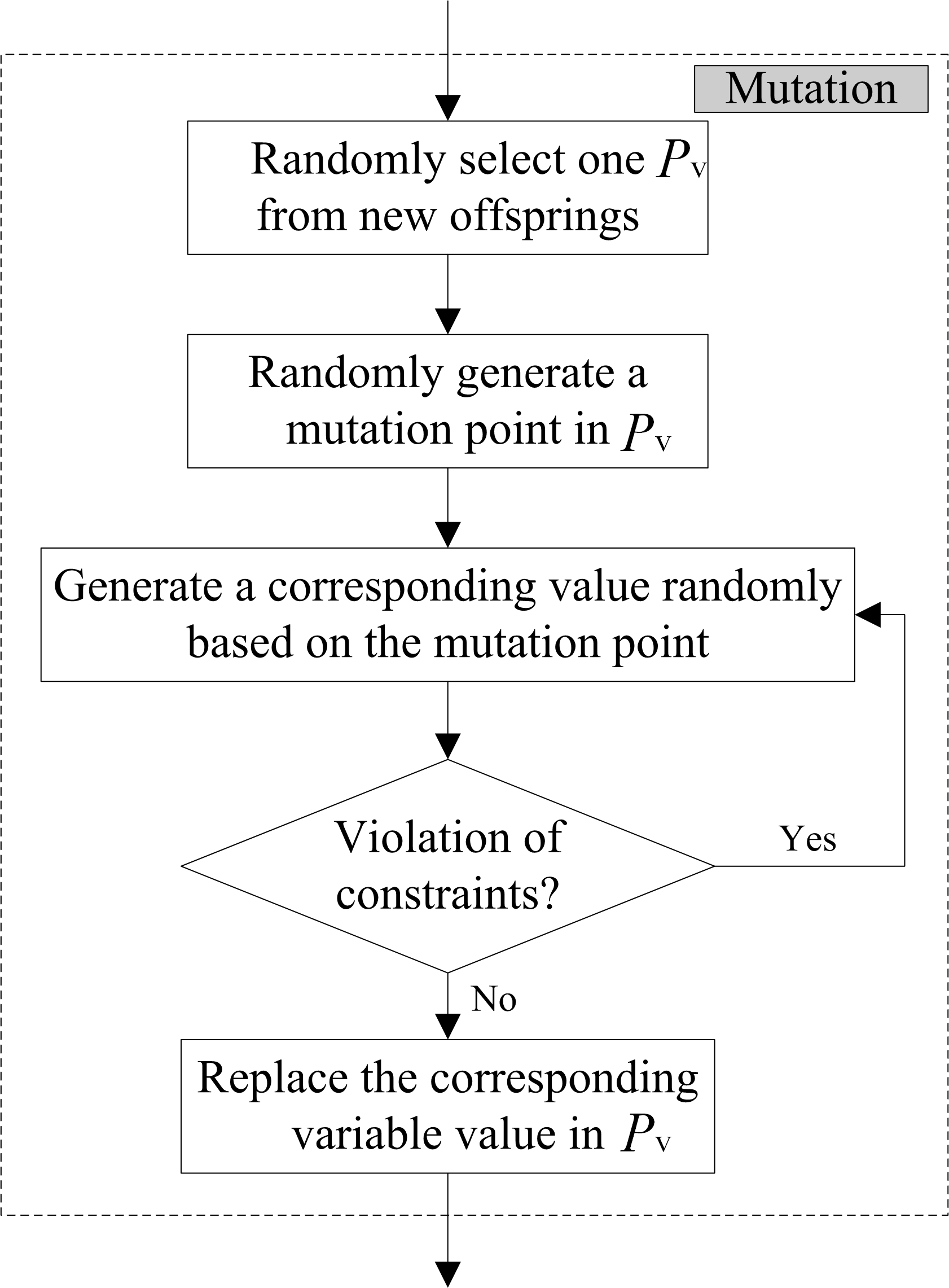


**Figure 5.** Mutation flowchart for CTPP primer design. When a mutation operation is performed, a mutation point in *P_v_* will be randomly selected and a corresponding value generated. Then the variable will be checked for violation of a constriction. If a constriction is violated, a random point is reselected and the variable is regenerated, otherwise the original value is replaced by the variable and the process continues in the next step.


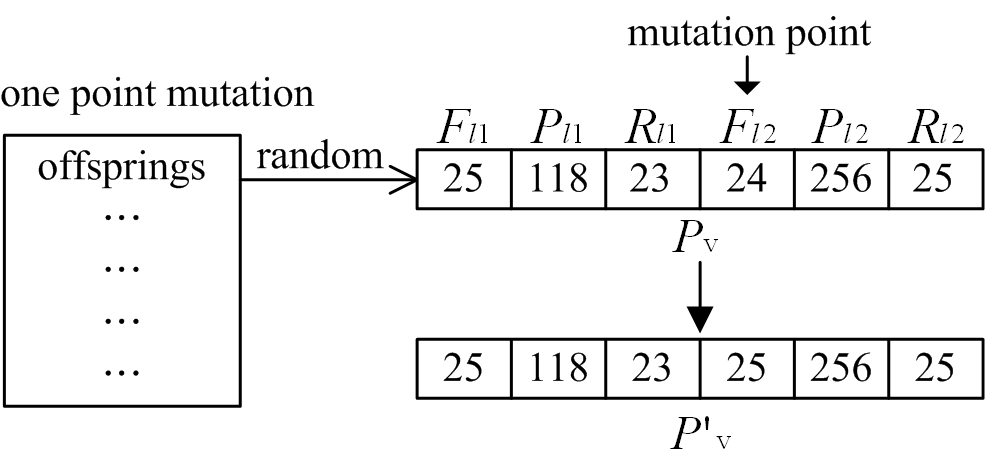


**Figure 6.** An example of a mutation operation for CTPP primer design. At the beginning, a *P_v_* = (25, 118, 23, 24, 256, 25) is randomly selected from the offsprings. Then one mutation point of the position *F_l_*_1_, *P_l_*_1_, *R_l_*_1_, *F_l_*_2_, *P_l_*_2_ or *R_l_*_2_ is randomly generated. In our example the position the mutation point is *F_l_*_2_. Then a random value between 16 and 28 of *F_l_*_2_ is generated. This value replaces the corresponding variable value in *P_v_*. Finally, the new offspring *P*'*_v_* = (25, 118, 23, 25, 256, 25) is generated.

1. ***Online web-based tool providing***

The original conference paper does not provide the web-based tool. It describes a blemish in an otherwise perfect thing is the graphical user interface (GUI) is still developing and constructing.

The web-based GA-CTPP tool with a user-friendly interface had been provided for academic users in the manuscript. It is available at <http://bio.kuas.edu.tw/ga-ctpp/availability.jsp>.

1. ***Native English revised***

The manuscript had been proofread by native English; however, the original conference paper does not.

Summary of comparison the original conference paper with the manuscript:

| **Differences** | **the original conference paper** | **the manuscript** |
| --- | --- | --- |
| data set | Janus kinase 2 gene (30 SNPs) | SLC6A4 gene (288 SNPs) |
| fitness function | lack the complete descriptions | provide the calculation of the fitness values for primer constraint functions |
| wet experiment validation | No | Yes |
| parameter settings | not a standard parameter settings | 1. standard parameter settings based on DeJong and Spears’ parameter settings  2. a increased population size parameter to 1000 |
| flowchart and examples | No | Yes  1. GA-based CTPP primer design  2. crossover flowchart for CTPP primer design  3. an example of a crossover operation for CTPP primer design  4. mutation flowchart for CTPP primer design  5. an example of a mutation operation for CTPP primer design |
| web-based tool providing | No | Yes, it is available at <http://bio.kuas.edu.tw/ga-ctpp/availability.jsp> |
| Native English revised | No | Yes |

*Reference:*

1. Yang CH, Cheng YH, Chuang LY, Chang HW: **Genetic Algorithm for the Design of Confronting Two-Pair Primers**. *Ninth IEEE international Conference on BioInformatics and BioEngineering (BIBE)* 2009:242-247.
2. Sambrook J, Fritsch EF, Maniatis T: **Molecular cloning**: Cold Spring Harbor Laboratory Press Cold Spring Harbor, NY; 1989.
3. De Jong KA, Spears WM: **An analysis of the interacting roles of population size and crossover in genetic algorithms**. In*.*, vol. 1: Springer; 1990: 38–47.
