# Supplementary material for: Confronting two-pair primer design for enzyme-free SNP genotyping based on a genetic algorithm
Source: BMC Bioinformatics. 2010 Oct 13;11:509. doi: 10.1186/1471-2105-11-509 (PMC2964683; doi:10.1186/1471-2105-11-509)
Supplement: Additional file 2 — 'The performances for primer design using our proposed GA-CTPP algorithm between different population sizes of Dejong and Spears's parameter settings'. [file 1471-2105-11-509-S2.DOC]

**Additional file 2.** The performances for primer design using our proposed GA-CTPP algorithm between different population sizes of Dejong and Spears’s parameter settings

|  | Constraints | | | | | | | | | |
| --- | --- | --- | --- | --- | --- | --- | --- | --- | --- | --- |
| population size | primer length difference | GC% | GC clamp | *T*_m_ | *T*_m_ difference | product length | dimer | hairpin | specificity | average fitness |
| 50 | 649/864 | 1107/1152 | 645/1152 | 998/1152 | 204/864 | 615/864 | 2752/2880 | 990/1152 | 1117/1152 | 469.70 |
| 100 | 661/864 | 1114/1152 | 638/1152 | 1042/1152 | 269/864 | 595/864 | 2771/2880 | 954/1152 | 1125/1152 | 441.27 |
| 200 | 674/864 | 1110/1152 | 642/1152 | 1051/1152 | 318/864 | 594/864 | 2758/2880 | 985/1152 | 1118/1152 | 413.09 |
| 300 | 640/864 | 1118/1152 | 643/1152 | 1079/1152 | 348/864 | 593/864 | 2749/2880 | 997/1152 | 1119/1152 | 396.78 |
| 400 | 690/864 | 1110/1152 | 634/1152 | 1084/1152 | 382/864 | 601/864 | 2765/2880 | 959/1152 | 1121/1152 | 382.78 |
| 500 | 677/864 | 1109/1152 | 646/1152 | 1086/1152 | 408/864 | 607/864 | 2753/2880 | 973/1152 | 1126/1152 | 376.92 |
| 600 | 674/864 | 1107/1152 | 645/1152 | 1086/1152 | 439/864 | 606/864 | 2754/2880 | 964/1152 | 1124/1152 | 370.80 |
| 700 | 681/864 | 1112/1152 | 645/1152 | 1103/1152 | 460/864 | 609/864 | 2756/2880 | 980/1152 | 1127/1152 | 352.08 |
| 800 | 681/864 | 1109/1152 | 640/1152 | 1082/1152 | 459/864 | 601/864 | 2752/2880 | 979/1152 | 1122/1152 | 349.64 |
| 900 | 662/864 | 1111/1152 | 654/1152 | 1087/1152 | 485/864 | 616/864 | 2732/2880 | 956/1152 | 1125/1152 | 339.64 |
| 1000 | 684/864 | 1115/502 | 653/1152 | 512/1152 | 608/864 | 752/864 | 2760/2880 | 969/1152 | 1121/1152 | 331.05 |
